# Focused Modulation of Brain Activity: A Narrative Review

**DOI:** 10.3390/biomedicines13081889

**Published:** 2025-08-03

**Authors:** Aisha Zhantleuova, Altynay Karimova, Anna P. Andreou, Almira M. Kustubayeva, Rashid Giniatullin, Bazbek Davletov

**Affiliations:** 1 Department of Biophysics, Biomedicine and Neuroscience, Al-Farabi Kazakh National University, Almaty A15E3C7, Kazakhstan; aisha.zhantuleyova@kaznu.edu.kz (A.Z.); altaneuro2022@gmail.com (A.K.); almira.kustubaeva@kaznu.edu.kz (A.M.K.); 2 Headache Research, Wolfson Centre for Age-Related Diseases, Institute of Psychiatry, Psychology and Neuroscience, King’s College London, London SE1 1UL, UK; anna.andreou@kcl.ac.uk; 3 Neuresta, Inc., San Diego, CA 91991, USA; 4 A.I. Virtanen Institute for Molecular Sciences, University of Eastern Finland, 80101 Kuopio, Finland; rashid.giniatullin@uef.fi; 5 Department of Biomedical Science, University of Sheffield, Sheffield S10 2JA, UK

**Keywords:** neuromodulation, deep brain stimulation, transcranial electrical stimulation, transcranial magnetic stimulation, focused ultrasound stimulation, chemogenetics, magnetogenetics, optogenetics, toxins

## Abstract

A wide range of strategies have been developed to modulate dysfunctional brain activities. This narrative review provides a comparative analysis of biophysical, genetic, and biological neuromodulation approaches with an emphasis on their known or unknown molecular targets and translational potential. The review incorporates data from both preclinical and clinical studies covering deep brain stimulation, transcranial electrical and magnetic stimulation, focused ultrasound, chemogenetics, optogenetics, magnetogenetics, and toxin-based neuromodulation. Each method was assessed based on specificity, safety, reversibility, and mechanistic clarity. Biophysical methods are widely used in clinical practice but often rely on empirical outcomes due to undefined molecular targets. Genetic tools offer cell-type precision in experimental systems but face translational barriers related to delivery and safety. Biological agents, such as botulinum neurotoxins, provide long-lasting yet reversible inhibition via well-characterized molecular pathways. However, they require stereotaxic injections and remain invasive. To overcome individual limitations and improve targeting, delivery, and efficacy, there is a growing interest in the synthesis of multiple approaches. This review highlights a critical gap in the mechanistic understanding of commonly used methods. Addressing this gap by identifying molecular targets may help to improve therapeutic precision. This concise review could be valuable for researchers looking to enter the evolving field of the neuromodulation of brain function.

## 1. Introduction

A growing variety of neuromodulation strategies are used to interrogate and modify brain activity across experimental and clinical settings. These approaches can be broadly categorized into biophysical, genetic, and biological methods—each with distinct mechanisms of action and translational challenges. In this narrative review, a critical comparative analysis of the current neuromodulation techniques, evaluating their molecular specificity, safety, reversibility, and translational potential, is provided. Emphasizing mechanistic clarity may help guide future combinations of methods, improve therapeutic design, and support the development of more targeted neuromodulatory strategies. This perspective may be particularly valuable for researchers aiming to choose their direction within the evolving field of neuromodulation.

## 2. Biophysical Methods

**Figure 1:**
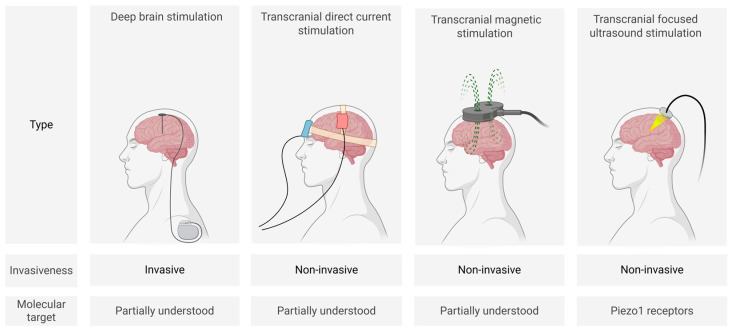
Schematic representation of main biophysical neuromodulation techniques (created in BioRender, Zhantleuova, A. (2025), https://BioRender.com/q3pnqqn) (accessed on 23 July 2025).

Biophysical methods (Figure 1) for modulating neuronal activity include invasive deep brain stimulation (DBS) as well as non-invasive techniques such as transcranial direct current stimulation (tDCS), transcranial magnetic stimulation (TMS), and focused ultrasound stimulation (FUS). These approaches are currently the most clinically established neuromodulation strategies.

### 2.1. Deep Brain Stimulation

Deep brain stimulation is an invasive neuromodulatory technique that was approved by the US Food and Drug Administration (FDA) nearly three decades ago (Table 1). It involves the surgical implantation of bipolar electrodes (typically 1.27 mm in diameter and 1.5 mm in height) into specific subcortical brain regions to deliver electrical pulses (commonly 1–3.5 V, 60–210 µs, and 130–185 Hz for voltage, pulse width, and frequency, respectively) [1,2]. The applied electric field, controlled by the patient, affects neuronal activity by influencing the opening and closing of voltage-gated ion channels, thereby modulating neuronal excitability. In addition to ion channel modulation, DBS affects key neurotransmitter systems, including dopaminergic, glutamatergic, and GABAergic pathways. It also influences non-neuronal elements of the brain microenvironment, such as glial and endothelial cells, and is associated with long-term plasticity and neuroprotective effects, including the upregulation of neurotrophic factors like BDNF and GDNF [3].

DBS is a well-established standard of care for movement disorders such as Parkinson’s disease, essential tremor, and dystonia, and is currently under active investigation for a range of neuropsychiatric and neurodegenerative disorders, including treatment-resistant depression and Alzheimer’s disease [4,5,6]. It has also been used for pain conditions such as refractory cluster headache, with good outcomes [7,8].

The application of DBS is limited by its invasive nature, requiring stereotactic neurosurgery and long-term device management, leading to high costs. The economic burden is substantial: the average cost of the DBS device is approximately USD 21,500 ± 8900, the cost of surgery is USD 40,942.85 ± 17,987.43, and the total cost of treatment may reach USD 47,600 ± 23,000 during the first year of care [9]. Additional challenges include the risk of surgical complications, such as infection or hemorrhage, that could even lead to death, and the potential for adverse neuropsychiatric side effects, including mood alterations or cognitive changes [10,11,12,13].

Recent developments have aimed to overcome these limitations through adaptive deep brain stimulation (aDBS), a closed-loop system that dynamically adjusts stimulation parameters based on real-time neural biomarkers reflecting the patient’s condition. Technological progress in signal processing, artifact filtering, and neural data integration has contributed to more precise and energy-efficient stimulation [14]. Early trials demonstrated that personalized aDBS may lead to better motor outcomes and quality of life in patients with Parkinson’s disease compared to conventional open-loop DBS [15]. Although the full results have not yet been published, it was reported that approximately 98% of participants in the ADAPT-PD trial opted to continue with adaptive DBS rather than revert to conventional stimulation [16].

While DBS was initially conceptualized as a reversible functional lesion, it is now evident that its therapeutic effects extend well beyond simple neuronal inhibition. The technique reshapes network dynamics, modifies neurotransmitter turnover, and influences non-neuronal brain components, namely glial and endothelial cells. Emerging studies also point to intracellular signaling cascades and neurotrophic regulation as plausible molecular targets. Understanding molecular targets is essential for expanding clinical use and optimizing DBS.

**Table 1:** Clinical approval of neuromodulation methods.

Method	Clinical Indication	Target Brain Region	Approval Year
Deep brain stimulation	Essential tremor, Parkinsonian tremor	Ventral intermediate nucleus of thalamus	1997 [17]
	Parkinson’s disease	Internal globus pallidus, subthalamic nucleus	2002 [18], 2025 [19]—aDBS ^1^
	Primary dystonia (under HDE ^2^)	Internal globus pallidus, subthalamic nucleus	2003 [20]
	Obsessive–compulsive disorder (under HDE ^2^)	Anterior limb of the internal capsule	2009 [21]
	Epilepsy	Anterior nucleus of thalamus	2018 [22]
Transcranial direct current stimulation	Major depressive disorder	Prefrontal cortex	2015 [23]
	Chronic pain syndromes such as fibromyalgia and migraine	Primary motor cortex	2016 [24]
Transcranial magnetic stimulation	Major depressive disorder	Cerebral cortex	2008 [25,26], 2021 [27]—with comorbid anxiety
	Headache (migraine with aura)	Occipital cortex	2013 [28]
	Obsessive–compulsive disorder	Prefrontal cortex	2017 [29]
	Smoking cessation	Prefrontal cortex, insula	2020 [30]
Transcranial focused ultrasound stimulation	Essential tremor	Ventral intermediate nucleus of the thalamus	2016 [31]
	Parkinson’s disease	Ventral intermediate nucleus of the thalamus	2018 [32]—tremor; MRgFUS ^3^ 2021 [33]—mobility, rigidity, or dyskinesia; MRgFUS ^3^

^1^ aDBS—adaptive deep brain stimulation; ^2^ HDE—Humanitarian Device Exemption; and ^3^ MRgFUS—magnetic resonance image-guided focused ultrasound stimulation.

### 2.2. Transcranial Direct Current Stimulation

Transcranial direct current stimulation is a non-invasive procedure in which electrodes are placed on the scalp to deliver low-intensity electrical currents (typically 1–2 mA, and up to 4 mA under controlled conditions [34]) that modulate neuronal activity. The most common electrode sizes range from 25 to 35 cm^2^, producing current densities of approximately 0.28–0.80 A/m^2^, and stimulation is typically applied for 20–40 min, depending on the protocol [35].

Transcranial direct current stimulation allows for the modulation of neuronal firing via excitatory or inhibitory mechanisms depending on the stimulation polarity. Specifically, anodal stimulation influences the resting membrane potential toward depolarization, whereas cathodal tDCS shifts it toward hyperpolarization [36]. These effects can persist for up to 90 min after a single session; longer-lasting effects are typically achieved only after repeated applications [37]. Synaptic mechanisms resembling long-term potentiation and long-term depression have been proposed as contributors to the after-effects of tDCS [38].

Two-directional stimulation has shown benefits for the treatment of patients with major depressive disorder [39]. Frontal lateralization has been extensively studied using fMRI and EEG measurements of brain activity during the resting state and cognitive task performance [40,41,42]. Depressed patients are characterized by hypoactivity of the left dorsolateral prefrontal cortex (DLPFC) and hyperactivity of the right DLPFC. For that reason, opposite stimulation of the left and right DLPFC helps to balance brain activity between hemispheres [43,44]. Despite the fact that tDCS has shown efficacy in treating patients with acute depression, it has demonstrated little to no benefit in treatment-resistant depression. Assuming that DBS of the subgenual gyrus (BA 25) has yielded effective results for such patients [45], technical advancements in the spatial resolution of tDCS could offer potential advantages. tDCS has been shown to offer therapeutic benefits for patients with treatment-resistant epilepsy [46,47], migraine, and children and adolescents with attention-deficit hyperactivity disorder [48]. Patients with Parkinson’s disease may benefit from tDCS in improving cognitive functions, even if motor function remains unchanged [49]. In Parkinson’s disease rat models, tDCS has been shown to alleviate depression-like behaviors [50].

The integration of neuromodulation techniques with neuroimaging may provide efficacy information simultaneously (online) or post stimulation (offline). In particular, the combination of tDCS with functional near-infrared spectroscopy (fNIRS) delivered an evaluation of cortical hemodynamic responses to neurostimulation. The effects of neuromodulation were evaluated via changes in EEG rhythms in patients after stroke during rehabilitation. An increasing number of integrated neuroimaging and neuromodulation (fNRIS-tDCS, EEG-tDCS) studies confirmed the significant impact of neurostimulation on brain activation [51,52].

A major strength of tDCS is its non-invasive nature, which confers a favorable safety profile compared to invasive neuromodulatory approaches. Additionally, the simplicity and portability of the technique enable its implementation in home-based environments, thereby enhancing its feasibility for widespread use. From an economic perspective, tDCS remains one of the most cost-effective neuromodulatory interventions. Hospital-based tDCS programs for depression have been estimated at EUR 1555.60 per patient [53]. Although not approved by the FDA, tDCS has received approval in the European Union for conditions such as depression and chronic pain (Table 1).

Nevertheless, the clinical efficacy of tDCS remains limited by several technical and biological factors. A major constraint is its low spatial resolution and diffuse current distribution, which is strongly influenced by electrode size, placement, and inter-electrode distance. To address this, newer electrode montages have been developed. For instance, high-definition tDCS utilizes small circular electrodes (~1 cm diameter) in a 4 × 1 ring configuration, significantly improving the focality of stimulation compared to conventional pad montages [54]. Furthermore, some researchers have proposed 4 mA protocols or individualized electric field dosing to enhance clinical effects, especially in patients who may be underdosed at standard 2 mA settings [34]. However, these approaches introduce their own challenges. Higher intensities increase the risk of scalp discomfort, burning sensations, and may raise concerns about safety in self-administered contexts. Moreover, electric field personalized dosing often requires MRI scans, complicating home-based implementation [55].

Compounding these issues, a wide range of different stimulation protocols have been employed, making it even more challenging to conclude on its actual therapeutic efficacy [56]. While tDCS remains the primary focus here, transcranial electrical stimulation also encompasses transcranial alternating current stimulation, transcranial random noise stimulation, and transcranial pulsed current stimulation, each of which modulates neural activity via distinct types of currents.

Ongoing research continues to explore the molecular mechanisms underlying tDCS to refine biomarkers of responsiveness and develop personalized protocols that can improve reproducibility and clinical outcomes across diverse populations.

### 2.3. Transcranial Magnetic Stimulation

Transcranial magnetic stimulation is a technique that uses rapidly changing magnetic fields to induce small electric currents that can modulate the activity of the underlying cortex. The main types of TMS are single-pulse TMS (sTMS), which produces a single magnetic pulse per application, and repetitive TMS (rTMS), which produces multiple magnetic pulses per application.

Clinically, sTMS delivered via a portable, FDA-approved (Table 1), hand held device has been shown to be effective in alleviating migraine attacks [57], while its long-term use has been shown to have preventive effects in reducing the frequency severity of migraines [58]. Preclinically, it has been shown to reduce cortical excitability and the induction of cortical spreading depression [59,60]. Interestingly, it has also been shown to modulate the excitatory activity of third-order trigeminothalamic neurons through the modulation of GABAergic activity that suppresses corticothalamic inputs [60].

rTMS can produce both inhibitory and excitatory effects depending on the frequency and pattern of stimulation. rTMS typically runs at 10 Hz; however, low-frequency TMS (LF-TMS; ≤1 Hz) is also widely used and typically induces inhibitory effects [61]. Inhibitory LF-TMS protocols have been applied in both research and clinical settings to modulate hyperactive brain regions associated with psychiatric and neurological disorders. For example, stimulation of the right DLPFC has been shown to reduce cortical excitability and is associated with antidepressant effects [62]. Similarly, LF-TMS of the left temporoparietal cortex has been used to alleviate auditory hallucinations in schizophrenia, with meta-analyses reporting significant reductions in symptom severity [63]. Clinical studies also report that LF-TMS applied to the right DLPFC can reduce panic-related symptoms in patients with panic disorder [64]. A meta-analysis showed that LF-TMS applied to the right DLPFC was as effective as high-frequency (10–20 Hz) rTMS applied to the left DLPFC in treating major depressive disorder; however, LF right-sided rTMS produced fewer side effects and had less risk of seizures [65]. Furthermore, brain oscillation-synchronized stimulation using real-time EEG-triggered TMS shows efficacy in neuromodulation [66]. Real-time EEG-TMS, by providing continuous effects of stimulation, may help to optimize protocol depending on the ongoing individual’s brain functional state. In addition, integrated fNIRS with TMS provides quantification of stimulation effects [67]. Both online (real-time) and offline (post hoc) measurements of responses to TMS were considered part of a “closed-loop” system. Recent engineering has produced other rTMS modalities, such as theta-burst TMS with the typical theta burst stimulation protocol running at 50 Hz every 200 ms. A protocol of intermittent theta burst stimulation has been FDA-approved (Table 1) for treating major depressive disorder in adults [68,69,70].

Despite the vast number of clinical applications showing therapeutic effects, TMS efficacy is limited by its low spatial resolution, shallow depth of penetration, interindividual variability, and the need for repeated sessions to maintain therapeutic effects. Treatment efficacy is strongly correlated with the number of cumulative sessions, and protocols often require 20–30 daily sessions, with some extending to 50 sessions, each costing approximately USD 300, although this cost varies widely by region [71,72].

TMS with neuronavigation significantly improves spatial resolution compared to traditional TMS. Neuronavigation, using real-time brain imaging, enables the precise targeting of specific brain regions in MNI space and allows for a more accurate delivery of stimulation. For instance, precise rTMS of the left DLPFC has shown clinical efficacy, and its effectiveness was found to depend on subgenual connectivity, assessed via resting-state functional MRI [73].

Future efforts should focus on refining stimulation protocols and improving the accuracy of cortical targeting by incorporating complementary techniques. Additionally, a deeper understanding of the molecular pathways affected by TMS may support the discovery of predictive biomarkers and facilitate the development of targeted neuromodulation.

### 2.4. Focused Ultrasound Stimulation

Low-intensity transcranial-focused ultrasound is a non-invasive neuromodulation technique that delivers focused ultrasound waves through the skull to modulate neuronal activity within specific brain regions [74]. The acoustic waves used in FUS typically operate within a frequency range of 250–700 kHz, allowing for the modulation of both cortical and deep subcortical structures with spatial precision in the range of 1–5 mm [75]. The intensity of the acoustic energy remains below 100 W/cm^2^ [76].

FUS has shown promise in preliminary clinical studies for modulating mood and emotional networks, and improving conditions such as chronic pain, minimal consciousness state, and drug-resistant temporal lobe epilepsy [77]. This technique, approved by the FDA for the treatment of essential tremor and Parkinson’s disease (Table 1), recently showed promising results in clearing amyloids from the brain in patients with Alzheimer’s disease [78]. FUS can facilitate the penetration of monoclonal antibodies, such as an anti-Alzheimer’s drug aducanumab, into brain tissue, resulting in the clearance of amyloids plaques [79]. Since FUS can lead to some changes in local temperature, the modern approach suggests simultaneous thermometry [80]. Even though the primary target cells for FUS are not fully established, the beneficial action of FUS is currently related to the opening of the blood–brain barrier (BBB) [81,82]. Recently discovered highly mechanosensitive Piezo1 channels were proposed as the main molecular targets of FUS [83,84].

A key limitation of FUS is imprecise targeting due to skull variability, which could be solved by a combination of FUS with imaging techniques (magnetic resonance image-guided focused ultrasound stimulation or MRgFUS). Acoustic coupling through hair also poses challenges, though recent studies show that using oil as a coupling medium can improve effectiveness and patient comfort without requiring hair removal [85].

### 2.5. Multi-Physical-Factor Stimulation Techniques

Given the limitations of single-modality neuromodulation methods in terms of spatial precision, penetration depth, and temporal specificity, there is growing interest in approaches that combine multiple physical modalities. One such technique is transcranial magnetoacoustic stimulation (TMAS), which integrates ultrasound waves with static magnetic fields. TMAS can achieve millimeter-level targeting even in deep brain regions, offering up to a tenfold better focus compared to conventional stimulation [86]. TMAS has shown promise in preclinical models, such as improving cognitive performance in mice with Parkinson’s disease [86]. Additionally, combining different biophysical techniques—for example, cathodal tDCS with low-frequency TMS—provided stronger and more sustained inhibitory effects in motor cortical excitability, highlighting the therapeutic potential of multi-modal neuromodulation strategies [87].

Although not a neuromodulation technology per se, recent developments in high-resolution brain–computer interfaces (BCIs) such as those from Neuralink and Synchron demonstrate the potential for future integration with modulatory systems. In the ongoing PRIME Study, Neuralink’s fully implantable device, featuring a skull-mounted chip and flexible electrode threads, has enabled paralyzed individuals to control digital interfaces with high precision through cortical activity alone [88]. Meanwhile, Synchron’s endovascular BCI, implanted via blood vessels, has shown promising results in early clinical trials, offering a less invasive alternative [89]. While the current application is limited to neural decoding and external device control, the underlying platform offers a foundation for future bidirectional systems that could combine recording with targeted stimulation, thus blurring the boundary between BCIs and therapeutic neuromodulation. However, the deployment of such high-risk intracranial devices has raised legal and ethical concerns regarding patient protection, especially in light of limited recourse mechanisms following device-related harm. A recent policy analysis proposed the establishment of a no-fault compensation framework to address these gaps, emphasizing the need for regulatory tools that balance innovation with the protection of patients in the context of experimental neurotechnology [90].

## 3. Genetic Methods

**Figure 2:**
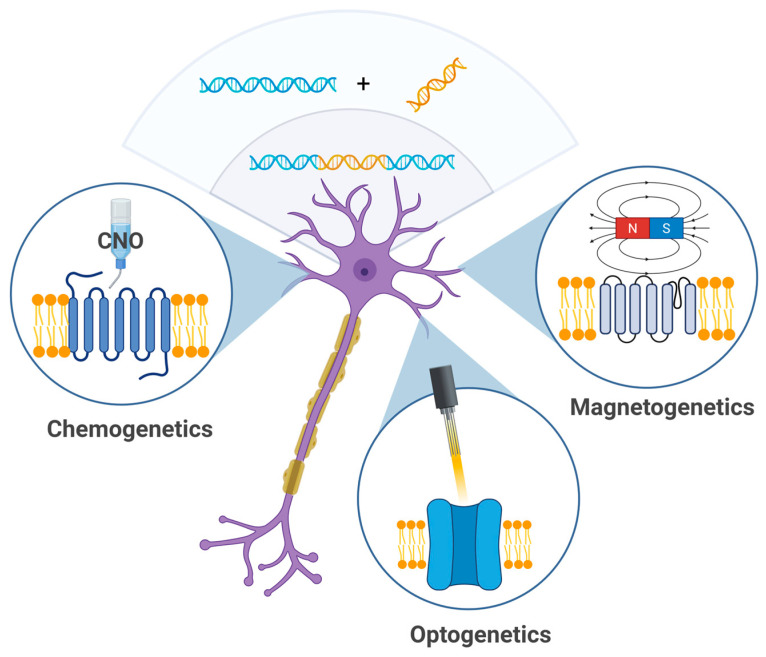
Genetic techniques—chemogenetics, optogenetics, and magnetogenetics—allow for modulation of neuronal activity using chemical agents (e.g., clozapine-N-oxide), light, or magnetic particles (created in BioRender, Zhantleuova, A. (2025), https://BioRender.com/fsp8kcp).

Genetic neuromodulation (Figure 2) techniques aim to achieve high cellular specificity by introducing genetically encoded, stimulus-sensitive protein receptors into neural tissue using viral vectors or similar gene delivery technologies. Upon successful transduction, these molecules can be selectively activated by specific external stimuli such as designer ligands (chemogenetics), magnetic fields (magnetogenetics), or light (optogenetics). While these methods have revolutionized preclinical neuroscience, their translational potential remains constrained. A major limitation lies in the need for the genetic manipulation of target brain cells, which raises significant concerns regarding health, safety, and ethical acceptability in human applications. Genetic neuromodulation strategies remain far from clinical implementation and require significant technological advances before broader application can be implemented.

### 3.1. Chemogenetics

Chemogenetics relies on genetically introduced effectors that are selectively responsive to specific ligands and induce a physiologically or biochemically meaningful response upon ligand binding [91]. The most widely used chemogenetic tool is DREADDs (Designer Receptors Exclusively Activated by Designer Drugs), which are activated exclusively by synthetic ligands such as clozapine-N-oxide (CNO). DREADDs offer several benefits: they do not require specialized equipment or invasive procedures for activation, and their ligands can penetrate deep brain regions. For instance, inhibitory DREADDs (e.g., hM4Di) have been successfully used to reduce excessive neuronal activity in the CA1 region of the mouse hippocampus. This suppression of neural activity not only alleviated hyperactivity, but also mitigated Alzheimer’s disease-like pathology in preclinical animal models [92]. Despite their advantages, DREADDs present certain limitations, since effective stimulation requires high doses of ligands. CNO is now known to undergo reverse metabolism into clozapine—a compound with broad affinity for endogenous receptors. This conversion contributes significantly to DREADD activation, but also introduces off-target effects [93]. To address these concerns, next-generation ligands such as deschloroclozapine (DCZ) have been developed [94]. At low doses, DCZ showed high specificity without off-target effects in DREADD-naïve primates performing cognitive tasks [95]. Importantly, the use of viral vectors to stereotaxically deliver and express designer receptors poses safety challenges, potentially limiting their therapeutic applicability in clinical settings. To date, no DREADD-based system has entered human trials, and additional work is required to develop safer ligands and targeted delivery systems for future applications.

### 3.2. Magnetogenetics

Magnetogenetics, the magnetic control of genetically targeted cells, has developed into two mechanistically distinct approaches. One strategy utilizes magnetic nanoparticles to deliver localized mechanical or thermal stimuli that activate mechano- or thermosensitive ion channels such as Piezo1 or TRPV1, enabling the precise control of cell signaling without ligand–receptor interactions. The second approach involves the expression of magnetically responsive proteins, such as the electromagnetic perceptive gene (EPG), which allows specific neuronal populations to respond to external magnetic fields. For instance, the activation of EPG in inhibitory interneurons has been shown to reduce seizure frequency in rodent models [96]. Both approaches offer non-invasive modulation with deep tissue penetration, but they face significant limitations, including unclear molecular mechanisms (particularly in EPG-based systems), reliance on head-mounted resonant coils, technical challenges in generating safe and effective alternating magnetic fields for whole-body applications, and limited temporal resolution [97]. The use of viral vectors to deliver magnetically responsive proteins also brings about safety challenges, potentially limiting their therapeutic applicability in clinical settings.

### 3.3. Optogenetics

Optogenetics is a set of techniques that use light in the visible spectrum to control the functional activity of cells through light-sensitive proteins, such as opsins, whose genes are introduced into the biological system in advance. The delivery and expression of opsin effectors can be achieved either through transient expression in specific neuronal populations using viral vectors carrying opsin genes or through stable expression in the brains of transgenic animals [98]. Optogenetic tools have enabled neuroscientists to manipulate the activity of neurons and glial cells with high temporal and spatial precision in experimental animals. For instance, the optogenetic inhibition of targeted axonal projections from the lateral orbitofrontal cortex (lOFC) to the striatum in mice reduced spiking in medium spiny neurons and alleviated obsessive–compulsive behavior, confirming the critical role of the lOFC–striatal pathway in this disorder [99]. Another study demonstrated that the optogenetic inhibition of mouse striatal GABAergic neurons increased microvessel density and growth factor expression in the peri-infarct region [100]. In a therapeutic context, the optogenetic inhibition of the subthalamic nucleus by Yoon et al. led to significant improvement in forelimb akinesia in a unilateral mouse model of Parkinson’s disease [101]. However, this method has some limitations when considering its use both in preclinical research and as a potential human therapeutic. Overexpression of microbial opsins in neural tissue can adversely affect neuronal physiology, and light activation may cause phototoxicity. The need for complex fiber-optic devices that must be securely attached to the animal’s skull is another drawback. Additionally, the precise control of optogenetic stimulation intensity can be challenging, especially due to the heterogeneous expression of opsins.

## 4. Biological Methods

**Figure 3:**
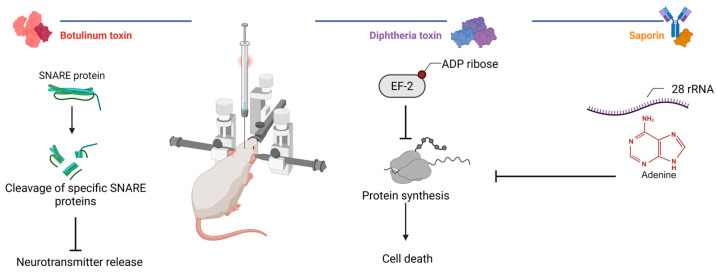
Mechanisms of action of selected toxins in experimental neuromodulation. Botulinum toxins cleave neuronal SNARE proteins to inhibit neurotransmission, whereas diphtheria toxin and saporin inhibit ribosomal protein synthesis (created in BioRender, Zhantleuova, A. (2025), https://BioRender.com/igz5iqh).

Biological neuromodulation approaches (Figure 3) involve the use of natural or engineered toxins to selectively inhibit neuronal activity following a local injection. These methods are primarily applied in preclinical research and rely on invasive intracerebral delivery to achieve focal effects. A key advantage of this approach lies in the well-characterized molecular mechanisms of toxin agents. Depending on the molecular construct, the effect can be irreversible or reversible, lasting from days to several months. This flexibility enables both the temporary silencing and permanent ablation of defined neuronal populations. However, the necessity of stereotactic injections and the potential for off-target distribution remain important limitations. Despite these challenges, biological methods hold translational promise when combined with refined targeting strategies and delivery systems.

### 4.1. Protein Synthesis Inhibiting Toxins: Saporin and Diphtheria Toxin

Toxins that inhibit protein synthesis, such as saporin and diphtheria toxin (DT), are widely used in experimental neuroscience to achieve the irreversible silencing of defined neuronal populations. These agents act by disrupting the translational machinery, leading to apoptotic cell death. Saporin is a type I ribosome-inactivating protein that depurinates a specific adenine residue within 28S rRNA, thereby halting ribosomal activity and protein synthesis [102]. DT, in contrast, enters cells via receptor-mediated endocytosis and delivers its catalytic domain into the cytosol, where subsequent ADP-ribosylation of elongation factor 2 (EF-2) effectively blocks translational elongation, thereby inducing cell death [103].

Modified saporin can be targeted to specific neurons via genetic fusion with cell-specific ligands. For example, 192IgG-saporin, an antibody conjugate targeting cholinergic neurons, has been used to selectively ablate cholinergic neurons in the basal forebrain [104]. This approach induces cognitive impairments, including deficits in memory and learning [105,106], mimicking the cholinergic dysfunction observed in Alzheimer’s disease. In Parkinson’s disease modeling, the targeted delivery of saporin conjugated to a monoclonal antibody to the dopamine transporter in the left striatum or lateral ventricle of adult male rats resulted in selective dopaminergic neuron destruction in the ipsilateral substantia nigra, with no off-target effects [107]. The local injection of saporin conjugated with quantum dots into the substantia nigra led to dopaminergic neuron loss, microglial activation, and impaired motor coordination, providing a model of Parkinson’s disease [108]. Apart from neurodegenerative disease models, saporin conjugates have been used to study sleep regulation. The targeted elimination of orexin 2 receptor-expressing neurons in the hypothalamus using orexin–saporin induces narcolepsy-like behavior [109], while their ablation in the substantia nigra or the ventrolateral preoptic nucleus caused insomnia [110,111].

A recent study used native DT to investigate the role of glutamatergic neurons in the sub-laterodorsal tegmental nucleus; neuronal ablation abolished REM sleep and enhanced fear memory [112]. Various modifications of diphtheria toxin have been developed to selectively target specific neuronal populations. For example, to model Alzheimer’s disease, a nerve growth factor–diphtheria toxin conjugate was used to deplete basal forebrain cholinergic neurons [113]. The development of a DT-urotensin-II fusion toxin [114] allowed for the selective ablation of cholinergic neurons in the pedunculopontine tegmental nucleus, revealing their limited role in reward processing, learning, or locomotion [115,116,117]. Tf-CRM107, a conjugate of transferrin and a mutated DT, showed promise in treating malignant brain tumors, although systemic toxicity limited its potential [118]. Other studies incorporated DT constructs targeting glioblastoma cells, achieving significant tumor regression in preclinical models [119,120].

### 4.2. Neurotransmission Inhibiting Toxins: Botulinum Neurotoxins

Botulinum neurotoxins represent a class of biological agents with unique properties of reversible neuronal inhibition. Several BoNT serotypes (A-H) exist in nature, differing in their duration of inhibitory effects, ranging from weeks to months, due to their intricate mechanism of action in the presynaptic endings of neurons [121]. BoNTs exert their effects by blocking neurotransmitter release through the cleavage of SNARE proteins, preventing synaptic vesicle fusion [122]. This temporary inhibition of neurotransmission allows for the precise modulation of neuronal activity, enabling researchers to investigate both normal and pathological brain function.

The intracerebral administration of BoNTs has been extensively studied experimentally to model neurological disorders. Early studies demonstrated that BoNT/B injections into the entorhinal cortex led to cognitive deficits, impaired learning, and reduced long-term potentiation in aged rats [123]. Subsequent research showed that intracerebroventricular BoNT/A administration caused persistent memory retrieval impairments [124]. Intrastriatal BoNT/A injections in normal rats revealed minimal cognitive effects, though treated animals exhibited reduced anxiety [125]. BoNT has been used to either model or treat epilepsy preclinically, depending on the site of injection. While intrahippocampal BoNT/B infusion induced proconvulsant effects, including a reduced seizure threshold and spontaneous seizures [126], other studies demonstrated antiseizure properties when injected into the hippocampus or amygdala in the models of chemically induced epilepsy [127,128,129]. A modified botulinum molecule, BiTox, with reduced paralytic properties, was used to inhibit neuronal firing in the suprachiasmatic nucleus of the hypothalamus, highlighting the potential of re-engineered BoNTs to modulate specific brain circuits [130].

BoNT/A has shown therapeutic potential in the 6-OHDA rat model of Parkinson’s disease, where intrastriatal injections significantly reduced apomorphine-induced rotations [131,132]. The BoNT/A2 variant proved more effective than BoNT/A1, offering greater efficacy with fewer side effects [133,134]. Repeated BoNT/A injections produced stronger and longer-lasting effects, improving motor function, gait, and dynamic locomotion [135,136,137,138]. Additionally, BoNT/A demonstrated antidepressant-like effects, enhanced olfactory performance, and modulated receptor binding in the striatum by normalizing D_2_/D_3_ receptor availability and reducing D_1_ receptor binding [139,140,141,142,143,144]. Importantly, observational studies in human patients have shown that BoNT/A injected into the sphenopalatine ganglion can be effective in trigeminal neuralgia, cluster headaches, and migraines [145,146].

The stereotaxic injection of BoNT/E into the primary motor cortex of rats 24 h prior to middle cerebral artery occlusion provided neuroprotection in ischemic brain injury by inhibiting glutamate release and preventing neuronal loss [147]. In exploratory studies, the transient silencing of the striate cortex via BoNT/E injection at the peak of the critical period (postnatal day 14) led to impaired visual maturation, reduced acuity, and prolonged plasticity, with deficits persisting even after recovery [148]. Similarly, a BoNT/A recombinant construct, when delivered to the adult visual cortex, modulated visual function, highlighting the potential of botulinum neurotoxin for studying both brain development and functional plasticity in adulthood [149]. The injected volume (1 μL) led to toxin spread confined to a volume of less than 1 mm^3^ from the injection site, providing a useful measure for future interventions. The absence of neuronal loss has been confirmed in histological studies following intrastriatal BoNT/A injection [150]. Interestingly, BoNT/A exhibited bidirectional axonal and transsynaptic transport, with cleaved SNAP-25 detected in multiple brain regions. This distribution depended on the distance from the injection site and connectivity to the striatum, and botulinum-cleaved SNAP-25 persisted for at least a year [151].

Recent advances in bioengineering have led to the development of chimeric botulinum molecules targeting specific neuronal subpopulations. These constructs typically combine the catalytic light chain of botulinum neurotoxins with customized receptor-binding domains. One example is the opioid receptor-targeted botulinum molecule, Dermorphin-Bot (Derm-Bot) [152]. In preclinical studies, this engineered construct selectively silenced nociceptive neurons and produced long-lasting analgesia in mouse models of chronic pain, without affecting motor function or causing systemic toxicity. Following intrathecal administration, Derm-Bot inhibited neurons in the spinal cord, expressing µ-opioid receptors and producing analgesic effects similar to conventional opioids, but at doses nearly a thousand times lower. Derm-Bot effectively alleviated mechanical hypersensitivity in inflammatory pain models induced by CFA injection into the ankle joint or hind paw. Further work demonstrated that a Substance P–botulinum conjugate, SP-Bot, could mediate the persistent silencing of pain pathways, with re-inducible effects upon repeated injection, providing a basis for long-term, reversible neuromodulation [153]. The Substance P part of the molecule allowed it to enter neurons that express neurokinin-1 receptors, which play a key role in transmitting pain signals in the spinal cord.

Together, these studies exemplify the potential of designer BoNT-based therapeutics to achieve the specific, reversible, and long-lasting inhibition of neuronal activity. However, their use remains limited by the need for invasive delivery and the potential for remote spread via transsynaptic transport, warranting the further refinement of delivery methods and safety assessment for clinical translation.

## 5. Future Directions

Despite decades of research and growing clinical applications, neuromodulation remains an evolving field with considerable conceptual and mechanistic gaps. While various techniques—including biophysical, genetic, and biological approaches—have demonstrated potential for modulating neural circuits, their therapeutic precision is still limited by an insufficient understanding of molecular targets and intracellular signaling pathways. Our comparative analysis (Table 2) reveals a consistent trade-off between the advantages and disadvantages of each neuromodulation technique.

Despite significant advances in basic research, some of these technologies are still far from clinical translation, primarily due to issues related to biosafety, delivery complexity, and insufficient mechanistic understanding. Genetic methods—such as optogenetics, chemogenetics, and magnetogenetivs—offer unparalleled cell-type specificity and well-defined intracellular mechanisms, making them powerful tools for dissecting neural circuits. However, their clinical utility is hampered by several barriers: the requirement for viral vector delivery, the risk of immune responses, the cytotoxic potential of magnetic nanoparticles, and ethical concerns associated with long-term genetic modification. Moreover, preclinical studies increasingly reveal that these technologies may induce off-target and systemic effects, especially when applied at therapeutic dosages. Future directions should focus on enhancing the safety of these tools through improved vector engineering and the development of cell-type-specific ligands. Minimally invasive delivery strategies, such as systemic administration with targeted transport across the BBB, may hold promise for reaching deep brain structures without invasive procedures.

In parallel, biological toxins offer an alternative route to targeted neuronal inhibition with defined intracellular action, such as the disruption of SNARE proteins or ribosomal function. Botulinum toxins A and B have been approved for clinical use to treat many bodily functions, but not brain-specific applications. Currently, their application remains limited by delivery challenges and concerns regarding off-target transport. Furthermore, unlike other techniques, these agents do not allow for real-time modulation or adaptation to dynamic neural states, making them less suited for flexible or responsive therapeutic designs. By designing chimeric toxins with tailored binding and catalytic domains, researchers can achieve higher specificity and reversibility, enabling cell-type-selective silencing with minimal off-target effects.

In contrast, biophysical neuromodulation techniques have already reached clinical application in several psychiatric and neurological disorders. However, these methods often suffer from poor spatial specificity, shallow penetration, and most notably, a lack of clearly defined molecular targets. As a result, their therapeutic effects remain empirical, with high inter-individual variability and limited predictability. Moreover, although generally considered safe, the long-term effects of repeated stimulation remain poorly understood. Potential delayed or cumulative adverse effects—such as structural, metabolic, or immunological changes—have not been systematically evaluated in most clinical protocols, raising concerns about their widespread use without robust long-term safety data. Future efforts should focus on hybrid stimulation paradigms that integrate multiple physical modalities to enhance efficacy. For example, FUS-mediated opening of the blood–brain barrier may facilitate the targeted delivery of designer neurotoxins, genetic vectors, or nanocarriers to deep brain structures.

Critically, future innovation must emphasize the identification and validation of molecular targets—such as ion channels, neurotransmitter systems, and mechanosensitive proteins like Piezo1—that mediate the effects of neuromodulatory interventions. Defining these targets will not only improve mechanistic understanding, but also support the rational design of combined or synergistic therapies. Integrating molecular insights with multimodal neuromodulation platforms will be key to developing personalized treatment strategies. Further studies of the molecular mechanisms of neuromodulation methods, in addition to a deeper understanding of their long-term action, provide prospects for a possible beneficial combination of methods. Multimodal integration can help improve therapeutic outcomes by using the advantages of some methods and leveling their disadvantages with the help of other methods, or mutually enhancing the desired effect by combining methods.

## 6. Limitations

This review does not provide a systematic summary of all available neuromodulation studies. Instead, it adopts a narrative format, deliberately focusing on the comparative evaluation of mechanistic specificity, safety, and translational feasibility across the main representative techniques. No formal inclusion or exclusion criteria were applied; rather, studies were selected to illustrate conceptual advances, clinical relevance, or unresolved mechanistic issues.

While the general advantages and limitations of neuromodulation strategies have been addressed in previous reviews, the distinctive contributions of this work lie in its wider coverage of neuromodulation approaches and emphasis on molecular targets highlighting the potential translational significance of mechanistic precision. Additionally, the review draws attention to underexplored intersections between modalities that are often overlooked in conventional frameworks. This perspective may inform future mechanistic and translational research, particularly where neuromodulation remains empirically guided.

## Figures and Tables

**Table 2:** Comparison of advantages and disadvantages of neuromodulation methods.

Method		Advantage	Disadvantage
Biophysical	Deep brain stimulation	Adjustable and reversible effects	Invasive procedure with surgical risks; high financial cost and maintenance burden; potential side effects
	Transcranial direct current stimulation	Non-invasive; portable; safe	Low spatial resolution; shallow penetration; variable efficacy
	Transcranial magnetic stimulation	Non-invasive; protocol flexibility	Limited depth and spatial precision; high inter-individual variability; potential side effects
	Transcranial focused ultrasound stimulation	Non-invasive; high spatial precision; capable of deep brain targeting	Low temporal accuracy; early clinical stage
Genetic	Chemogenetics	High cell-type specificity; no need for external devices	Requires viral vector delivery; high ligand doses may cause off-target effects; low time precision
	Magnetogenetics	Good targeting precision	Unclear molecular mechanisms; technical complexity; requires genetic modification via viral vectors
	Optogenetics	Extremely high temporal and spatial precision	Requires genetic modification; light delivery via implanted optical fibers; potential phototoxicity; heterogeneous opsin expression
Biological	Protein synthesis-inhibiting toxins	Flexible targeting via conjugation with ligands or antibodies	Requires stereotaxic injection; irreversible neuronal loss
	Neurotransmission-inhibiting toxins	Reversible inhibition; modifiable duration	Requires stereotaxic injection; potential off-target transport

## Data Availability

Not applicable.

## Abbreviations

The following abbreviations are used in this manuscript:

6-OHDA	6-hydroxydopamine
ADAPT-PD	Adaptive DBS Algorithm for Personalized Therapy in Parkinson’s Disease
aDBS	Adaptive deep brain stimulation
ADP	Adenosine diphosphate
BBB	Blood–brain barrier
BCIs	Brain–computer interfaces
BDNF	Brain-derived neurotrophic factor
BoNTs	Botulinum neurotoxins
CFA	Complete Freund’s Adjuvant
CNO	Clozapine-N-oxide
DBS	Deep brain stimulation
DCZ	Deschloroclozapine
Derm-Bot	Dermorphin-botulinum neurotoxin
DLPFC	Dorsolateral prefrontal cortex
DREADDs	Designer Receptors Exclusively Activated by Designer Drugs
DT	Diphtheria toxin
EEG	Electroencephalogram
EF-2	Elongation factor 2
EPG	Electromagnetic perceptive gene
FDA	US Food and Drug Administration
fMRI	Functional magnetic resonance imaging
fNIRS	Functional near-infrared spectroscopy
FUS	Focused ultrasound stimulation
GABA	Gamma-aminobutyric acid
GDNF	Glial cell line-derived neurotrophic factor
HDE	Humanitarian Device Exemption
LF-TMS	Low-frequency transcranial magnetic stimulation
lOFC	Lateral orbitofrontal cortex
MRgFUS	Magnetic resonance image-guided focused ultrasound stimulation
MRI	Magnetic resonance imaging
rRNA	Ribosomal ribonucleic acid
rTMS	Repetitive transcranial magnetic stimulation
SNAP-25	Synaptosomal-associated protein 25
SP-Bot	Substance P–botulinum neurotoxin
sTMS	Single pulse transcranial magnetic stimulation
tDCS	Transcranial direct current stimulation
TMAS	Transcranial magnetoacoustic stimulation
TMS	Transcranial magnetic stimulation
